# Statins reduce all-cause mortality in chronic obstructive pulmonary disease: an updated systematic review and meta-analysis of observational studies

**DOI:** 10.18632/oncotarget.20304

**Published:** 2017-08-17

**Authors:** Wen-Feng Li, Yu-Qing Huang, Cheng Huang, Ying-Qing Feng

**Affiliations:** ^1^ Department of Cardiology, Guangdong Cardiovascular Institute, Guangdong Provincial Key Laboratory of Coronary Heart Disease Prevention, Guangdong General Hospital, Guangdong Academy of Medical Sciences, Guangzhou, 510080, China

**Keywords:** statins, chronic obstructive pulmonary disease, COPD, systematic review

## Abstract

Recently, a number of observational studies have suggested that use of statins reduces mortality in patients suffering from chronic obstructive pulmonary disease (COPD). To obtain a more valid assessment, we update the meta-analysis of the effect of statins on COPD exacerbation and mortality. We searched for eligible articles using PubMed, Medline, Embase, the Cochrane Central Register of Controlled Trials, Cochrane Databases and Web of Science between January 2006 and February 2017, with no restrictions. The hazard ratio (HR) with 95% confidence interval (CI) was estimated. Publication bias was evaluated by funnel plot and Begg's test. Sensitivity analyses were also conducted. Twenty studies with a total of 303,981 patients were included. Thirteen articles provided data on all-cause mortality (165,221 participants), and the pooled hazard ratio of 0.65 (95% CI: 0.57–0.74, *P* < 0.001). Nine cohorts involving 155,435 patients reported data for COPD exacerbation with or without hospitalization, and they gave a HR of 0.58(95%CI: 0.48–0.72, *P* < 0.001). Our systematic review of exclusively observational studies showed a clear benefit of statins for patients suffering from COPD.

## INTRODUCTION

Chronic obstructive pulmonary disease (COPD) is currently one of the leading causes of morbidity and mortality, the prevalence of this disease is rapidly increasing worldwide, and in 2020 it has been predicted that this disease will reach the third cause of mortality worldwide [1]. Also, COPD is a debilitating, irreversible disease with currently available therapies targeting symptom control and exacerbation reduction, and treatments that improve survival are needed [2]. Cardiovascular (CV) events are increasingly being recognized as a major cause of death in patients with diverse forms of lung disease, such as COPD [3]. At the same time, the vast majority of evidences have come from observational studies suggested that some cardiovascular drugs, such as statins, antiplatelet drugs, angiotensin-converting enzyme inhibitors (ACEI), angiotensin receptor blockers(ARB), and b-blockers have a beneficial effect on reducing mortality for COPD [4]. What is more, comorbidities may further complicate COPD, leading to increased hospitalizations, mortality and healthcare costs, and current therapies for comorbid diseases, such as statins, may provide unexpected benefits for COPD patients [5]. Statins are a class of cholesterol lowering drugs widely used for treatment of hypercholesterolemia and cardiovascular diseases. Statins have anti-inflammatory actions that modulate the innate immune system [6] and reduce inflammatory markers such as C-reactive protein and interleukin-6(IL-6) [7]. Most population-based observational studies have reported associations between statin use and a reduced of mortality and hospitalization among COPD patients [8–10]. There is a growing interest in determining whether statins improve the prognosis of patients with COPD. Systematic reviews prior to 2015 have suggested that statins are associated with a beneficial role in the treatment of COPD [11–14]. Since then, several additional trials have been conducted in this setting [15–19]. All in all, the results of some of the trials showed a beneficial effect, but the results were inconsistent when all trials were considered. We therefore conducted an updated systematic review and meta-analysis on the effect of statins on COPD exacerbation and mortality.

## RESULTS

### Studies retrieved and characteristics

**Figure 1:**
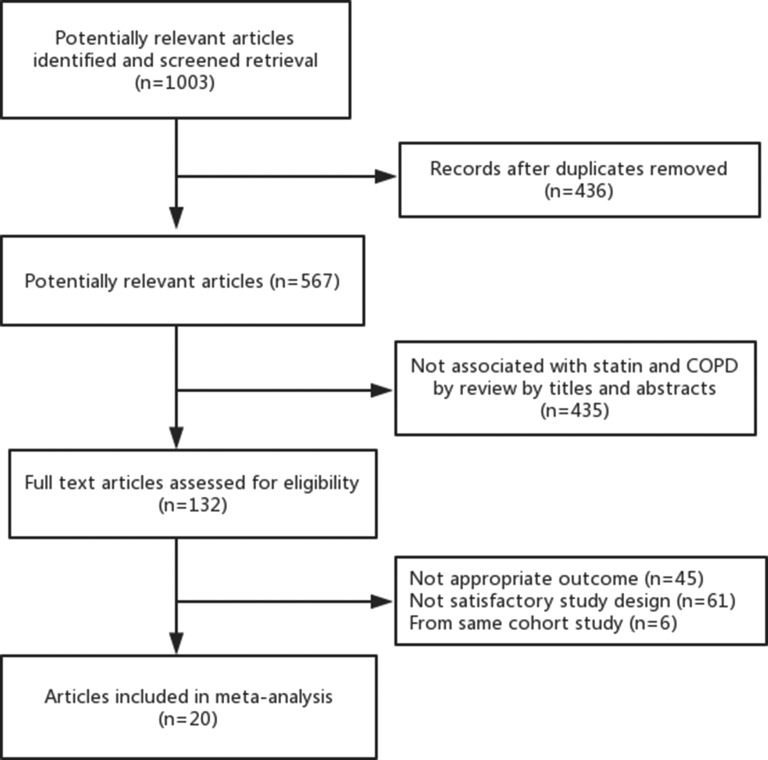
Flowchart of trial identification for meta-analysis

Overall our initial search found 1,003 articles that met the preliminary criteria. After removing duplicates, and screening titles and abstracts, 132 articles qualified for a full review. We finally found 20 eligible articles [3, 4, 7–10, 15–28] which included 10 prospective cohort studies and 10 retrospective cohort studies, comprising 303,981 individuals for this analysis (Figure 1). Supplementary Table 1 lists the baseline characteristics of the included studies. These 20 articles were published from 2006 to 2017. The number of patients in each cohort ranged from 107 to 103,004. The duration of follow-up ranged from three to 84 months. According to quality assessment criteria, the score of included articles was in the range 5 to 9, which showed moderate to high quality for all studies (Supplementary Table 2 and Supplementary Table 3). For the association between statins on COPD and risk of mortality, COPD exacerbation, hospitalization, and stroke, 15, 7, 5, and 1 studies, respectively, provided data. There were 13 cohorts reported all-cause mortality (*n* = 165,221), 3 reported COPD mortality (*n* = 128,445), 3 reported cardiovascular mortality (*n* = 4,425) and 2 reported cancer-mortality (*n* = 4,018). Also, there were 7 cohorts showed the effect of statins therapy on the risk of COPD exacerbation with or without hospitalization (*n* = 128,046), 5 cohorts showed the effect of statins therapy on the risk of hospitalization (*n* = 149,114) and 2 cohorts showed the effect of statins therapy on the risk of stroke (*n* = 1,717).

### All-cause and cause-specific mortality

**Figure 2:**
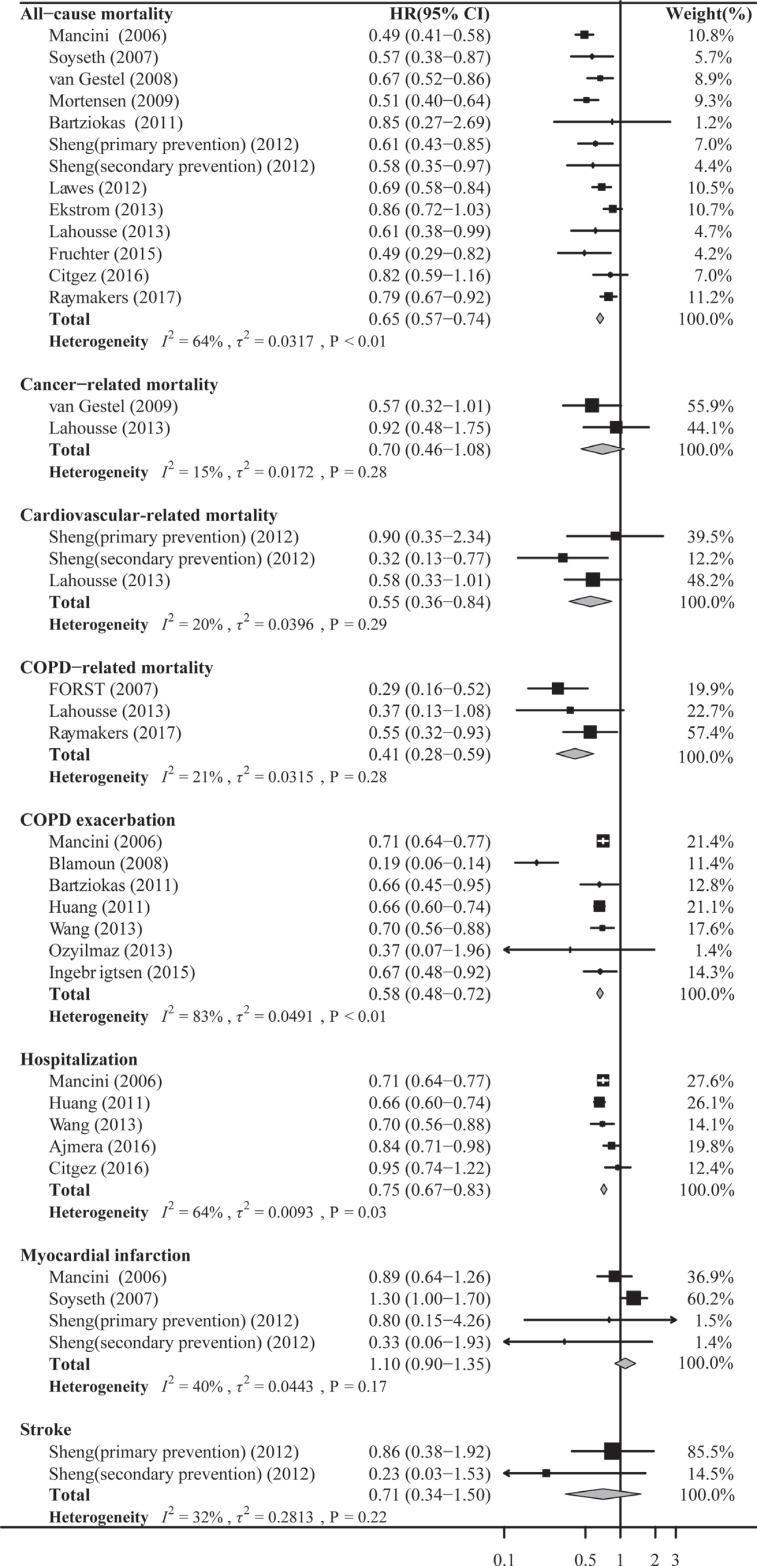
Forest plot showing effect of statins on all outcomes Pooled effect estimate is from a random fixed model.

There were 13 cohorts involving 165,221 patients reported data for the association between statins on COPD and risk of all-cause mortality. Random effects models analyses showed that significant association of statins use with a decrease risk of all-cause mortality (HR 0.65, 95%CI: 0.57–0.74; Figure 2), which with a significant moderate heterogeneity (*I*^2^ = 64%, *P* < 0.01). The analysis for three cohorts detected a protective effect of statin treatment on the risk of COPD mortality (HR 0.41, 95%CI: 0.28–0.59; Figure 2). The use of statins was associated with a significantly decrease risk of cardiovascular mortality (HR 0.55, 95%CI: 0.36–0.84; Figure 2). However, there was no significant association of statins treatment with a decrease risk of cancer mortality was observed (HR 0.70, 95%CI: 0.46–1.08; Figure 2).

### COPD exacerbation and hospitalization

There were 7 cohorts involving 128,046 patients reported data for the association between statins on COPD and risk of COPD exacerbation with or without hospitalization, and 5 cohorts involving 149,114 patients showed the effect of statins therapy on the risk of hospitalization. A random-model analysis for these cohorts evaluating HR of statin use to COPD exacerbation with or without hospitalization yielded a pooled HR of 0.58 (95%CI: 0.48–0.72; Figure 2) with a significant high heterogeneity (*I*^2^ = 83%, *P* < 0.01). Additionally, the analysis showed that statins use was associated with decrease COPD exacerbation requiring hospitalization (HR 0.75, 95%CI: 0.67–0.83; Figure 2).

### Stroke

There were 2 cohorts involving 1,717 patients from one article reported data for the association between statins on COPD and risk of stroke. The pooled HR for statins users versus statins non-users was 0.71(95%CI: 0.34–1.50; Figure 2).

### Sensitivity analysis

There was no evidence of publication bias based on visual inspection of funnel plots or based on Begg's or Egger's tests for each outcome (all *P* > 0.1). Sensitivity analyses confirmed that the association between statins on COPD and all outcomes did not change with the use of random effects models or fixed effects models for the meta-analysis. And when omitting one study at a time and recalculating the pooled HRs, we found the pooled result of all-cause mortality (Supplementary Figure 1) and COPD exacerbation with or without hospitalization (Supplementary Figure 2) still remained sturdy.

**Figure 3:**
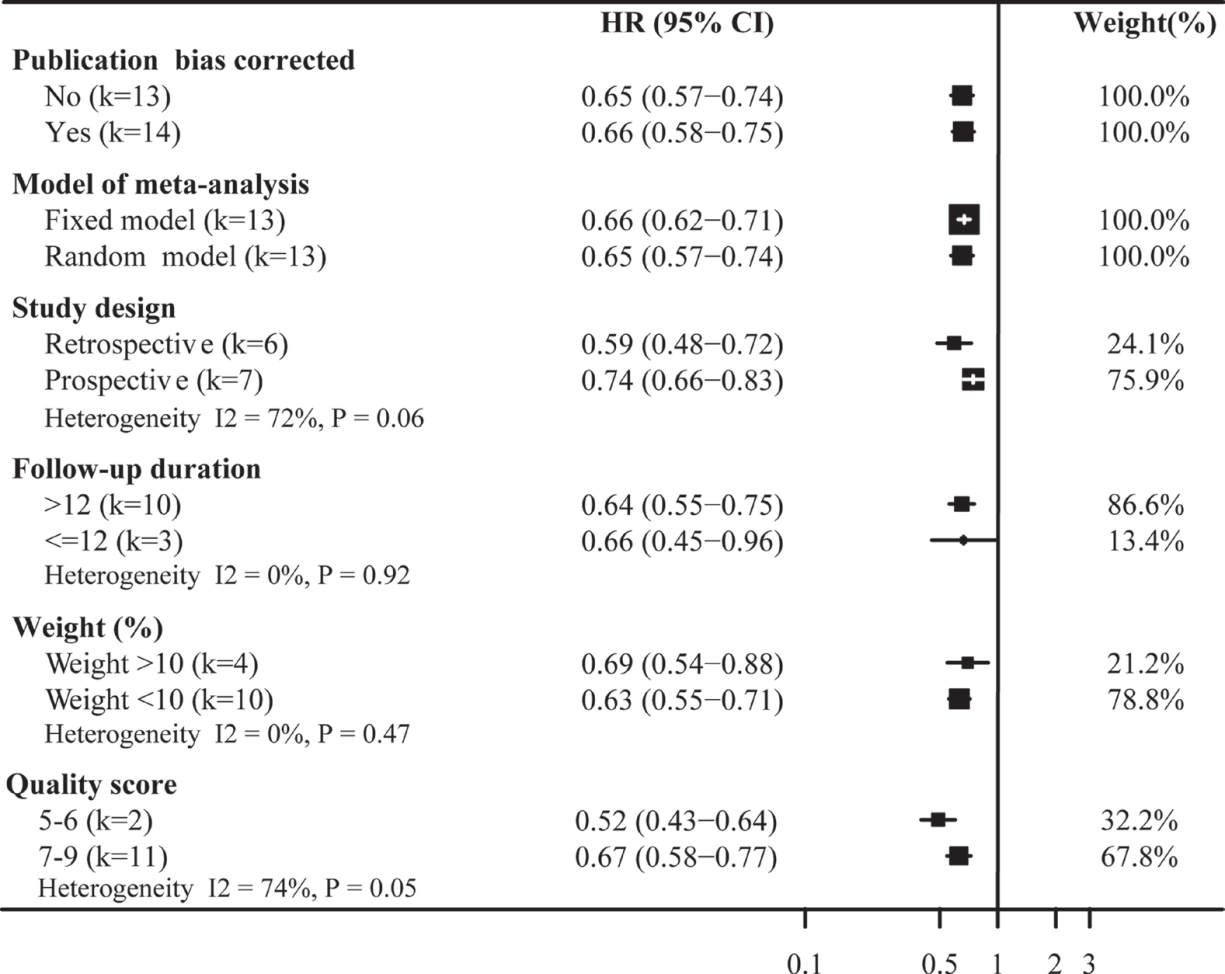
Sensitivity analysis of statins on all-cause mortality Pooled effect estimate is from a random fixed model. HR, hazard ratio.

**Figure 4:**
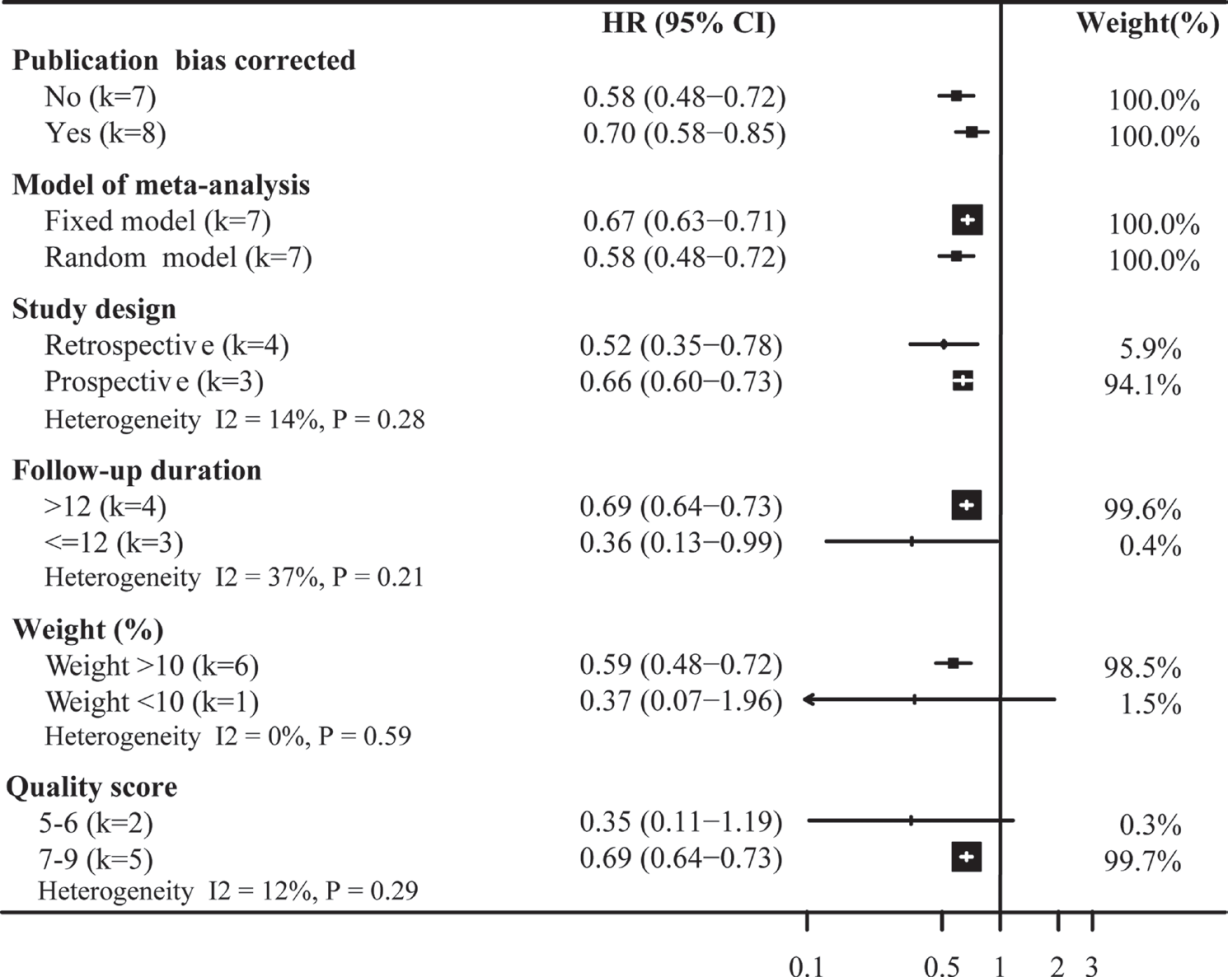
Sensitivity analysis of statins on COPD exacerbation Pooled effect estimate is from a random fixed model. HR, hazard ratio.

Figures 3 and 4 show the results of subgroup analyses for the risk of all-cause mortality and COPD exacerbation associated with statins on chronic obstructive pulmonary disease. The subgroup analyses for the risk of all-cause mortality associated with statins on COPD, we found the effect size from statins use was reduced in the prospective studies (HR 0.74, 95%CI: 0.66–0.83; Figure 3) and in the higher quality studies (HR 0.67, 95%CI: 0.58–0.77; Figure 3). Similarly, in Figure 4 the effect size conferred by statins appears to be decreased according to fixed model prospective design (HR 0.66, 95%CI: 0.60–0.73; Figure 4), bigger study size (HR 0.59, 95%CI: 0.48–0.72; Figure 4) and higher quality (HR 0.69, 95%CI: 0.64–0.73; Figure 4). Briefly, for all-cause mortality, study design and quality score were associated with strong heterogeneity, while the study weight and data source did not. For COPD exacerbation, we found no significant heterogeneity among subgroup comparisons.

## DISCUSSION

In the current systematic review and update meta-analysis, there were 303,981 cases of COPD from 20 articles, we found a beneficial, statistically significant association between statins treatment and COPD outcomes. Thirteen articles provided data on all-cause mortality (165,221 participants), and the pooled hazard ratio of 0.65 (95% CI: 0.57–0.74). Seven cohorts involving 128,046 patients reported data for COPD exacerbation, and they gave a HR of 0.58 (95%CI: 0.48–0.72). Statins use was associated with a 35% reduction in all-cause mortality (95% CI: 0.57–0.74) and a 59% reduction in COPD mortality (95% CI: 0.28–0.59). And the use of statins was associated with a significantly decrease risk of cardiovascular mortality (HR 0.55, 95%CI: 0.36–0.84). Additionally, the analysis showed that statins use was associated with a 42% lower risk of COPD exacerbation with or without hospitalization.

Based on the findings of our study, we showed a clear benefit of statins for patients suffering from COPD. The protective effect of statins could be explained by their pleiotropic effects, including inhibit vascular endothelial inflammatory response, stabilize athermanous plaque, antithrombotic effects, and improve endothelial function [29]. Chronic inflammation and oxidation reaction played vital role in the development of COPD and cardiovascular disease (CVD) [30]. At the same time, COPD, which consists of chronic bronchitis and emphysema, is a disease of the lungs, resulting from chronic inflammation and irreversible remodeling [21]. As COPD progresses, lots of inflammatory biomarkers, such as tumor necrosis factor-a, IL-1b, IL-8, and C-reactive protein were related to COPD [2]. These numerous inflammatory responses are believed to contribute to disease progression of COPD, and systemic inflammation often together with COPD and elevated circulating inflammatory factors can be an important risk factor for morbidity and mortality [31]. In theory, anti-inflammatory therapy should have a lot of protective effects on COPD due to the inflammatory nature of COPD. And statins is also a kind of anti-inflammatory drugs could beneficial effects of its on inflammation reduction. Similarly in COPD patients not taking statins, this pro-inflammatory effect would result in worse outcomes [32]. A large number of studies have demonstrated the ability of statins to modulate the immune responses and reduce inflammatory mediators including C-reactive protein, high sensitivity C-reactive protein, IL-6, IL-10, IL-8, and IL-17a levels, independent of the lipid lowering potential further supporting their potential use in inflammatory based diseases [33–35].

The findings from observational studies showed that statins reduced mortality or exacerbations in patients with COPD were not consistent with a large multi-center randomized controlled trial study [36]. The prospective randomized placebo-controlled trial of simvastatin in the prevention of COPD exacerbations (STATCOPE) as a randomized, controlled trial of simvastatin (at a daily dose of 40 mg) versus placebo, with annual exacerbation rates as the primary outcome [36]. There are several reasons that might be responsible for this conclusion. Firstly, the mean follow up time in this RCT was less than 2 years and contrasts with the longer follow up in the observational studies. Secondly, in that RCT there were strict exclusion criteria limiting this study to those with moderate to severe COPD and no cardiovascular risk. It has been estimated that this probably excluded as much as 60–80% of COPD patients who have underlying cardio-vascular risk and who might benefit from statin therapy [32]. In addition, in that RCT, the authors did not evaluate effect of statins in the form of HR or RR, which limit our quantitative analysis with this study. However, there was also a previous RCT which included healthy people with normal lipid levels but increased C-reactive protein levels showed that atorvastatin could significantly reduce C-reactive protein levels and the incidence of CV events [37].

The present study has a number of advantages over the previous meta-analysis; on the one hand, all of the published observational studies, included both comparative cross-sectional and cross-sectional studies in this meta-analysis up to now. On the other hand, the HR for COPD mortality or exacerbation and hospitalization was pooled across a large number of studies. What is more, the *P* value from the Egger's test was 0.05, implying that a publication bias could exist in the 20 studies conducted in patients with COPD. Of course, there were some limitations of the present study. Firstly, we only include observational studies for system analysis due to the few randomized controlled study on statins for COPD. Observational studies have relative lower clinical evidence than randomized controlled studies. Secondly, the crude (unadjusted) odds ratio or HR for statins in COPD patients was reported, as different studies used different set(s) of confounders. Thirdly, many of the confounding factors were not controlled in some of the studies included, and the sample size of several subgroups was small. In the end, due to different study have different sample size, year of publication, use of different kinds of statins and different follow-up time, therefore there were significant heterogeneities among the studies. But the consistency between the results from HRs by fixed model and random-model makes the results reliable despite the heterogeneity.

In conclusion, our systematic review and meta-analyses showed that statins use was associated with a reduction in all-cause mortality and COPD exacerbation or hospitalization. Our systematic review showed a clear benefit of statins for patients suffering from COPD. Although this meta-analysis and previous original studies have common limitations in their observational research, these studies have presented meaningful results. Therefore, there is needed more and more randomized, well-designed, multi-center, double blind clinical studies to identify the long term effects of statins in COPD.

## MATERIALS AND METHODS

### Search strategy and selection criteria

Following recommendations of the Meta-analysis of Observational Studies in Epidemiology group, [38] we searched electronic databases (PubMed, Medline, Embase, the Cochrane Central Register of Controlled Trials, Cochrane Databases and Web of Science) between January 2006 and February 2017, using the following search terms: “Hydroxymethylglutaryl-CoA Reductase Inhibitors” or “statin” or “statins” or “atorvastatin” or “cerivastatin” or “fluvastatin” or “pravastatin” or “pitavastatin” or “rosuvastatin” or “simvastatin” or “cerivastatin” or “mevastatin” and “COPD” or “chronic obstructive pulmonary disease” or “COAD” or “chronic obstructive airway disease” or “chronic obstructive lung disease” and “mortality” or “death” or “exacerbation” or “hospitalization” or “stroke” and “risk”. Studies were included for the current meta-analysis if they were prospective or retrospective cohort studies with statin therapy in patients with COPD; and they provided original data reporting adjusted relative risks and 95% confidence intervals (CI) for mortality or exacerbation or hospitalization or stroke associated with statin user or nonuser. In the case of multiple articles that were derived from the same cohort and reported the same associated events, we included only the latest published data in this analysis.

### Patient involvement

No patients were involved in setting the research question or the outcome measures, nor were they involved in developing plans for design or implementation of the study. No patients were asked to advice on interpretation or writing up of results. There are no plans to disseminate the results of the research to study participants or the relevant patient community.

### Data extraction and quality assessment

Two investigators independently identified the titles and abstracts of potentially suitable studies, and the information included participant number, age, sex, follow-up duration, characteristics of the exposure and the reported outcomes. If a study reported hospitalities at different time, we included the first follow-up period for analysis. We used the 9-star Newcastle-Ottawa quality assessment scale for quality assessment of cohort studies [39]. The scale is judged based on selection (four items, one star each), comparability (one item, up to two stars), and exposure/outcome (three items, one star each). In this analysis, we assigned quality as good (≥ 7 stars), fair (4–6 stars), and poor (< 4 stars).

### Statistical analysis

The data on the adjusted results were used for this meta-analysis. Heterogeneity among potentially studies was firstly assessed with the Q statistic, if *P* ≥ 0.10 was considered to indicate no significant heterogeneity. If no heterogeneity exits in the pooled result, results of random and fixed models are the same, and if significant heterogeneity is present, a random model is more conservative. And we used *I*^2^ test to assess the grade of heterogeneity. The grade divided into four levels, included low (0–25%), mild (25%–50%), moderate (50%–75%) and high (≥ 75%) [40]. We used a funnel plot and Begg's test to assess the existence of publication bias [41]. For sensitivity analysis, we recalculated the pooled relative risk by omitting one study at a time. All analyses were performed with RevMan5.3 (Cochrane Collaboration, Copenhagen, Denmark) and Stata12.0 (Stata Corp LP, College Station, TX).

## SUPPLEMENTARY MATERIALS FIGURES AND TABLES





## Abbreviations

All abbreviations have been defined within the manuscript.
