# Supplementary material for: Statins reduce all-cause mortality in chronic obstructive pulmonary disease: an updated systematic review and meta-analysis of observational studies
Source: Oncotarget. 2017 Aug 17;8(42):73000–8. doi: 10.18632/oncotarget.20304 (PMC5641186; doi:10.18632/oncotarget.20304)
Supplement: Supplementary file 1 [file oncotarget-08-73000-s001.pdf]

Statins reduce all-cause mortality in chronic obstructive pulmonary disease: an updated systematic review and meta-analysis of observational studies

SUPPLEMENTARY MATERIALS

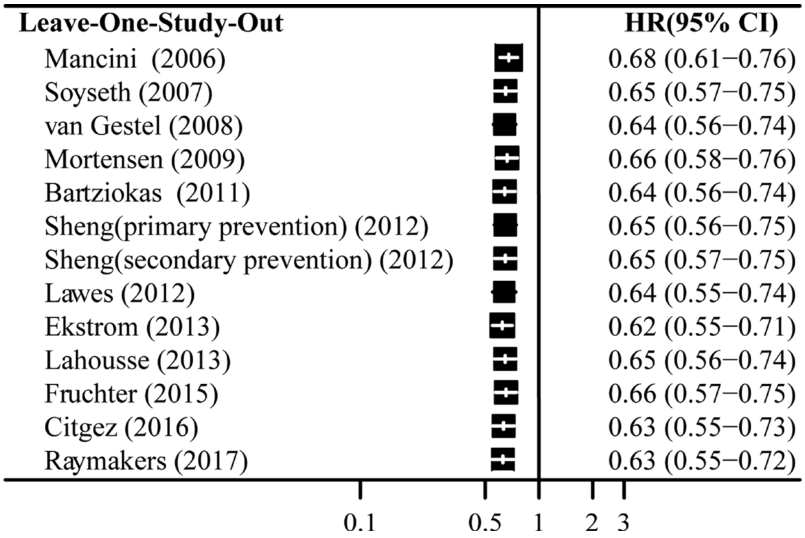

Supplementary Figure 1: Sensitivity analysis of statins on all-cause mortality. Pooled effect estimate is from a random fixed model. HR, hazard ratio.

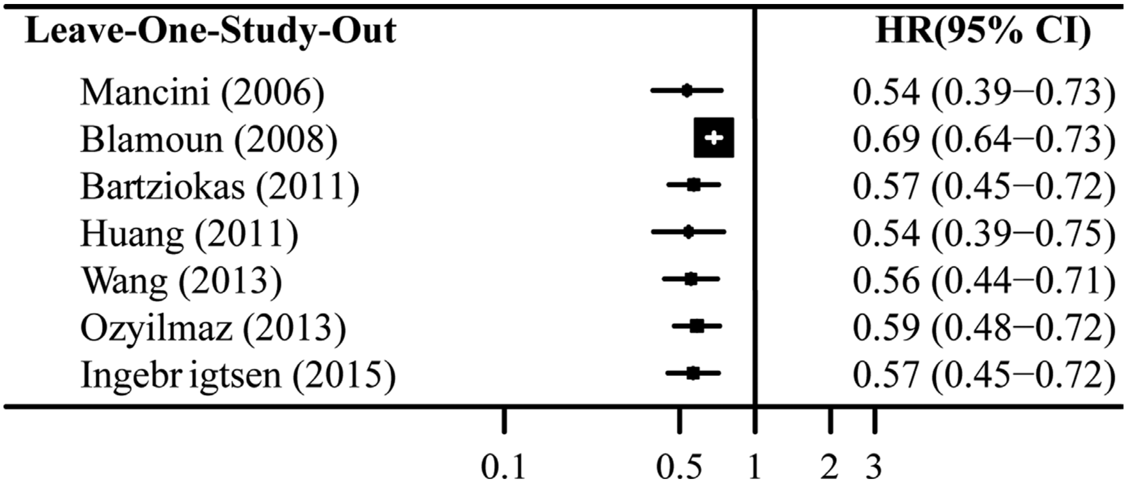

Supplementary Figure 2: Sensitivity analysis of statins on COPD exacerbation. Pooled effect estimate is from a random fixed model. HR, hazard ratio.

Supplementary Table 1: Summary of baseline characteristics of included trials

See Supplementary_Table_1

Supplementary Table 2: Quality assessment of cohort studies by Newcastle-Ottawa scale

Reference	Selection				Comparability	Outcome			Overall
	Representative of cases	Selection of controls	Ascertainment of exposure	Outcomes present at start of study	Comparability the design or analysis	Assessment of outcome	Adequate follow-up time (≥ 1 year)	Adequacy of follow up (≥ 80%)	Quality
van Gestel et al, 2008 [23]	★	–	★	★	★★	★	★	★	7
Sheng et al, 2012 [27]	★	★	★	★	★★	★	★	★	9
Ekström et al, 2013 [4]	–	–	★	★	★★	★	★	★	7
Lawes et al, 2012 [10]	★	–	–	★	★	★	★	★	7
Søyseth et al, 2007 [21]	★	–	–	–	★★	★	★	★	6
Bartziokas et al, 2011 [9]	★	–	★	★	★★	★	★	★	8
Lahousse et al, 2013 [7]	★	★	★	–	★	★	★	★	7
Mortensen et al, 2009 [24]	★	–	★	–	★	★	–	★	5
Frost et al, 2007 [20]	★	★	★	★	★	★	?	★	7
van Gestel et al, 2009 [25]	–	–	★	★	★	★	★	★	6
Blamoun et al, 2008 [22]	★	★	★	–	★	–	★	★	6
Huang et al, 2011 [26]	★	★	–	★	★	★	★	★	7
Fruchter et al, 2015 [15]	★	–	★	★	★	★	★	★	7
Ingebrigtsen et al, 2015[16]	★	★	★	–	★	★	★	★	7
Ajmera et al, 2016 [17]	★	★	★	★	★	★	★	★	8
Citgez et al, 2016 [18]	★	★	★	★	★★	★	★	★	9
Raymakers et al, 2017 [19]	★	★	★	★	★	★	★	★	8
Ozyilmaz et al, 2013 [28]	★	★	★	★	★	★	★	★	8

Supplementary Table 3: Quality assessment of nested case-control studies by Newcastle-Ottawa scale

Reference	Selection				Comparability	Outcome			Overall
	Definition of cases	Representativeness of cases	Selection of controls	Definition of controls	Comparability the design or analysis	Ascertainment of exposure	Same method for cases and controls	Non-Response rate	Quality
Mancini et al, 2006 [3]	★	★	★	★	★	★	★	★	7
Frost et al, 2007 [20]	★	★	★	★	★	★	★	★	8
Wang et al, 2013 [8]	★	★	★	★	★	★	★	★	8
