# Supplementary material for: Statins reduce all-cause mortality in chronic obstructive pulmonary disease: an updated systematic review and meta-analysis of observational studies
Source: Oncotarget. 2017 Aug 17;8(42):73000–8. doi: 10.18632/oncotarget.20304 (PMC5641186; doi:10.18632/oncotarget.20304)
Supplement: Supplementary file 2 [file oncotarget-08-73000-s002.docx]

| **Supplementary Table 1: Summary of baseline characteristics of included trials** | | | | | | |
| --- | --- | --- | --- | --- | --- | --- |
| Source | No of Participants | Study design | Age | Male | Statin user group | Outcomes |
| Forst et al,2007[[20](#_ENREF_20)] | 86,059 | A matched cohort and two separate case-control | NA | 39,716 in cohort;  4,943 in case-control | COPD patients were drawn from the Lovelace Patient Database | Pneumonia and COPD  mortality; Unspecified  pneumonia and influenza death |
| Soyseth et al ,2007[[21](#_ENREF_21)] | 854 | Retrospective cohort | 70.8 | 414 | Consecutive patients with a  diagnosis of COPD exacerbation at discharge from hospital | All-cause mortality |
| Blamoun  et al ,2008[[22](#_ENREF_22)] | 185 | Retrospective cohort | 69.7 for cases;  72.2 for controls | 119 | New patients admitted with a  diagnosis of COPD who had been  treated with statins | COPD exacerbations  Intubations secondary to COPD exacerbation |
| van Gestel  et al ,2008[[23](#_ENREF_23)] | 1,310 | Prospective cohort | 69 | 1,034 | Consecutive COPD patients underwent elective vascular surgery | All-cause mortality:  Short(30-day) and long-term (10-year) |
| Mortensen  et al ,2009[[24](#_ENREF_24)] | 11,212 | Retrospective national cohort | 73.5 For cases;  74.3 for controls | 10,993 | Subjects 65 years of age hospitalized with a COPD exacerbation | All-cause mortality |
| van Gestel et al ,2009[[25](#_ENREF_25)] | 1,310 | Prospective cohort | 69 for  Trials  69 for  controls | 1,034 | Consecutive COPD patients underwent elective vascular surgery | Cancer mortality |
| Bartziokas et al ,2011[[9](#_ENREF_9)] | 245 | Prospective cohort | 71.2 | 222 | Patients admitted to respiratory  medicine departments with a diagnosis of exacerbation of COPD | All-cause mortality;  Adverse outcomes index (death or need for mechanical ventilation);  COPD exacerbation |
| Huang et al ,2011[[26](#_ENREF_26)] | 18,721 | Nationwide population-based prospective cohort | 64 | 9,418 | Newly diagnosed COPD patients  who received statins for  hyperlipidemia treatment | Hospitalization for COPD exacerbation |
| Sheng et al ,2012[[27](#_ENREF_27)] | 1,274 for PP;  443 for SP | Prospective cohort | 68.5 for  trials,  68.7 for controls in PP;  70.2 for  trials,  74.9 for  Controls in SP | 619 in PP;  258 in SP | COPD patients in statin-exposed according to whether or not they were taking statin treatment during follow-up | All-cause mortality;  Total cholesterol;  Cardiovascular death;  Myocardial infarction;  Stroke |
| Ekstrom et al ,2013[[4](#_ENREF_4)] | 1,396 | Prospective cohort | 74.7 | 569 | Patients aged 45 Years or older started long-term oxygen therapy for physician diagnosed COPD | All-cause mortality |
| Lahousse  et al ,2013[[7](#_ENREF_7)] | 2,708 | Prospective cohort | 81 for  cases,  78 for  controls | 1,971 | COPD patients had received at least one prescription for statins between start and index date | All-cause mortality;  Cardiovascular mortality; Cancer |
| Ingebrigtsen et al ,2015[[16](#_ENREF_16)] | 5,784 | Retrospective cohort | 71 for  cases,  66 for  controls | 3,066 | COPD patients in statin-exposed according to whether or not they were taking statin treatment  during follow-up | COPD exacerbation |
| Ajmera et al ,2016[[17](#_ENREF_17)] | 19,060 | retrospective longitudinal dynamic cohort | NA | 7,726 | COPD patients in statin-exposed  according to whether or not they were taking statin treatment  during follow-up | hospitalizations and  emergency room and outpatient visits |
| Citgez et al ,2016[[18](#_ENREF_18)] | 795 | Prospective cohort | 68.2 for  trials,  67.6 for  controls | 486 | COPD patients had received a statin for at least 90 consecutive days | all-cause mortality;  hospitalization |
| Raymakers et al, 2017[[19](#_ENREF_19)] | 39,678 | Retrospective cohort | 71(11.6) | 32,906 | COPD patients had received a statin within 365 days | all-cause mortality; pulmonary mortality |
| Mancini et al ,2006[[3](#_ENREF_3)] | 103,004 | Population-based retrospective time-matched nested case-control | 77 | 43,605 | Patients were drawn from the  the Quebec Linked Databases: two distinct COPD cohort | All-cause mortality;  COPD hospitalization Myocardial infarction |
| Wang et al ,2013[[8](#_ENREF_8)] | 7,534 | Nationwide retrospective nested case- control | 74.6 for  cases; 74.1for controls | 6,044 | COPD patients were drawn from  the Longitudinal Health Insurance  Database | COPD exacerbation  requiring hospitalization |
| Fruchter et al ,2015[[15](#_ENREF_15)] | 615 | retrospective nested case- control | 71.8 | 421 | Consecutive patients with a diagnosis of COPD exacerbation | All-cause mortality |
| Ozyilmaz et al ,2013[[28](#_ENREF_28)] | 107 | Prospective cohort | 66.3 | 91 | Consecutive COPD patients who were admitted to out and inpatient  because of COPD exacerbation | COPD exacerbation |
| Lawes et al ,2012[[10](#_ENREF_10)] | 1,687 | Prospective cohort | 70.6 | 877 | Patients admitted to hospital with  a first primary hospital discharge  code consistent with COPD | All-cause mortality |

COPD, chronic obstructive pulmonary disease.
